# Functions of innate and acquired immune system are reduced in domestic pigeons (*Columba livia domestica*) given a low protein diet

**DOI:** 10.1098/rsos.150408

**Published:** 2016-03-23

**Authors:** Yuko Mabuchi, Theresa L. Frankel

**Affiliations:** Department of Physiology, Anatomy and Microbiology, School of Life Sciences, La Trobe University, Melbourne, Australia

**Keywords:** maintenance nitrogen requirement, phagocytosis, oxidative burst, lymphocyte proliferation, antibody production, parasite

## Abstract

Racing pigeons are exposed to and act as carriers of diseases. Dietary protein requirement for their maintenance has not been determined experimentally despite their being domesticated for over 7000 years. A maintenance nitrogen (protein) requirement (MNR) for pigeons was determined in a balance study using diets containing 6, 10 and 14% crude protein (CP). Then, the effects of feeding the diets were investigated to determine whether they were adequate to sustain innate and acquired immune functions. Nitrogen intake from the 6% CP diet was sufficient to maintain nitrogen balance and body weight in pigeons. However, the immune functions of phagocytosis, oxidative burst and lymphocyte proliferation in pigeons fed this diet were reduced compared with those fed 10 and 14% CP diets. Pigeons given the 6 and 10% CP diets had lower antibody titres following inoculation against Newcastle disease (ND) than those on the 14% CP diet. A confounding factor found on autopsy was the presence of intestinal parasites in some of the pigeons given the 6 and 10% CP diets; however, none of the pigeons used to measure MNR or acquired immunity to ND were infested with parasites. In conclusion, neither the 6 nor 10% CP diets adequately sustained acquired immune function of pigeons.

## 1. Introduction

Domestic pigeons (*Columba livia domestica*) are now found worldwide. They are kept for competition racing, showing of fancy breeds and food: they are also found in large numbers as feral birds especially in urban areas. Racing pigeons can travel vast distances during competitions and thus they have the potential to spread diseases [1,2].

As with many Columbidae species, they are granivorous and their diets are lower in protein than those of insectivores and omnivores [3,4]. Although recommended dietary crude protein (CP) content of feed for domestic pigeons has been reported as between 12 and 18%, this has not been determined experimentally and is based on reproduction of offspring [5] rather than maintenance requirements. Classical nutrition balance studies for determining maintenance nutrient requirements use a balance between nutrient intakes and losses and maintenance of body weight (BW); functioning of the immune system is seldom considered. The immune system in healthy birds requires a relatively small proportion of daily protein or amino acids to maintain it [6]. However, during immune system activation protein requirements increase and birds may go into negative nitrogen balance [7].

The immune system of birds consists of two components, the innate and the acquired (cellular-mediated or humoral) immune systems [8]. The innate immune system responds rapidly and gives rise to acute inflammatory responses; in comparison, the acquired immune system takes longer to respond to pathogens but it can result in inducible immune responses that are specific to particular antigens and have memory, e.g. previously vaccinated animals are able to protect themselves against subsequent exposures to pathogens [8].

Heterophils (the equivalent of mammalian neutrophils) are the most numerous of the leucocytes of the innate immune system. They respond rapidly to the entry of pathogens and have a variety of different mechanisms to overcome infective agents. The primary function of heterophils is to engulf foreign bodies and destroy them [9]. During the process of phagocytosis, pathogens are detected and bind to protein receptors on the heterophil surface [10], and this stimulates multicomponent signalling proteins and enzymes that can alter the formation of the actin cytoskeleton network so that the cell walls can move to engulf and sequester pathogens within the heterophil [11]. Once pathogens are phagocytized, cytoplasmic granules in the phagocyte come into contact with the entrapped pathogens. One process by which pathogens can be destroyed is termed oxidative burst, during which reactive oxygen species (ROS) can be produced [12]. The process is mediated by a multicomponent enzyme complex, involving protein kinase C (PKC) activated [9,13,14] by nicotinamide adenine dinucleotide phosphate (NADPH) oxidase [15]. A strong *in vitro* activator (mitogen) of the oxidative burst system in birds is phorbol myristate acetate (PMA) [9].

Should the innate immune system not overcome a pathogen that gains entry to the body then the various lymphocytes of the acquired immune system can be activated. Cellular-mediated immunity (CMI) involves T-lymphocyte proliferation, which is enhanced by release of cytokines [16]. These activate the humoral immune system as well as regulating innate immune responses [8]. The humoral immune system involves production of antibodies (glycoproteins) by B-lymphocytes with specific receptors for binding to particular antigens [8].

Besides circulating leucocytes, the immune system of birds consists of organs such as the thymus, spleen and bursa of Fabricius [8,17]. Birds do not possess lymph nodes, and therefore the Peyer's patches (Pps), lymphoid tissues located at various sites along the small intestine and consisting of primary and secondary lymphoid follicles, are very important [18]. They form a first line of defence against ingested bacteria and pathogens entering the body [18]. The Pps contain multiple cells such as macrophages, T- and B-lymphocytes, and therefore they have multiple immune functions by which they remove and kill pathogens.

Immune functions can be affected by many nutrients, and protein has been shown to be one of the most important nutrients to affect immune functions across many species, from insects, such as caterpillars (*Spodoptera littoralis*) [19] and African armyworms (*Spodoptera exempta*) [20], through fish (e.g. rainbow trout (*Oncorhynchus mykiss*) [21]), birds (e.g. poultry [22–29]), rodents (e.g. mice [30] and rats [31]) and people [32]. However, in birds most of this research has been carried out in growing poultry, and thus may not be applicable to adult racing or show pigeons.

A study on interactions between protein intakes and immunity of domestic pigeons could provide information that would be useful in accurately defining protein requirements that can maintain disease resistance. Therefore, this study using a cross-over nutrient balance experiment was designed to first determine endogenous nitrogen loss (ENL) and whether the three experimental diets containing 6, 10 and 14% CP satisfied maintenance nitrogen (protein) requirements (MNR) of young adult domestic pigeons (Experiment 1). Then, in Experiment 2, the three diets were used to firstly determine their effects on the innate immune functions of phagocytosis and oxidative burst using isolated heterophils, and then their effects on acquired immune responses were measured by lymphocyte proliferation and antibody production following inoculation with killed Newcastle disease vaccine (NDV). The effects of dietary protein levels on the surface areas of Pps were determined because of their importance in preventing pathogen entry from the gut. From the results, an assessment would be made of the dietary protein required to support immune function.

## 2. Material and methods

### 2.1. Pigeons, management and diets

Fifty-six mixed gender pigeons aged between six and nine months, purchased from a commercial breeder, were housed in aviaries 1.8 m long × 0.9 m wide × 1.8 m high. The aviaries were all in one shed, where temperature was maintained at 20–23°C with a light period between 07.30 and 19.30 h. When not given the experimental diets, pigeons were provided with Barastoc Pigeon Chips (Ridley AgriProducts, Pakenham, Victoria, Australia) at 10% of their BW.

Semi-purified experimental pelleted diets (table 1) were manufactured to order by Specialty Feeds (Glen Forest, Western Australia). The lowest dietary CP concentration of 6% was based on data for maintenance protein requirement of confined granivores (fig. 6.5 in [33]). The highest concentration, 14% CP, is used for many commercial diets, and 10% CP was intermediate between the other diets. The ingredients of each diet were as similar as possible except for the amount of starch and soy protein isolate (table 1; tables in the electronic supplementary material).

**Table 1.** The composition and calculated nutritional content of three semi-purified experimental diets for pigeons, containing different amounts of crude protein (CP) (data based on information provided by Specialty Feeds, Western Australia).

	dietary protein concentration		
ingredient	6%	10%	14%
starch	765	725	680
soya protein isolate	61	101	146
cellulose	50	50	50
soya bean oil	40	40	40
vitamins, minerals, amino acid premix	84	84	84
	calculated nutrient content		
protein, %	6.0	10.0	14.0
total fat, %	4.2	4.4	4.4
crude fibre, %	4.7	4.7	4.7
metabolizable energy, MJ kg^−1^	15	15	15
analysed N content (CP)^a^, %	1.05 (6.56)	1.88 (11.75)	2.59 (16.19)

^a^(CP) = crude protein calculated as N × 6.25.

### 2.2. Experiment 1. Determination of endogenous nitrogen loss and maintenance nitrogen (protein) requirements

Nitrogen intake and excretion were determined using a cross-over design so that each pigeon received each of the three experimental diets (6, 10 and 14% CP). Six pigeons were randomly chosen and housed two per aviary. Each experimental diet was provided at 35 g/day/pigeon for two weeks before a pigeon was placed in metabolism cages so that feed intake could be measured and excreta collected (details of the cages and methodology are in the electronic supplementary material). After 5 days in the metabolism cages the pigeons were returned to their aviaries and given a different experimental diet. Pigeons were weighed before and after being placed in a metabolism cage. Samples of uneaten food and excreta collected each day over 3 days were stored at −20°C then dried and analysed for nitrogen using a 2400 CHN/O analyser (series II PerkinElmer®, MA, USA). A factor of 6.25 was used to convert N to protein [34].

### 2.3. Experiment 2. Dietary protein intake and immune function

The remaining fifty pigeons were randomly assigned to a dietary (6, 10 and 14% CP) treatment group, 16 or 17 per group. Pigeons were weighed once a week.

Six weeks after the start of feeding experimental diets, 1 ml of blood taken from the wing veins was added to tubes containing EDTA (Vacuette®, Greiner Bio-One, Kremsmünster, Austria). Heterophils were isolated from peripheral blood by discontinuous gradients, (3 ml Histopaque®-1077 over 3 ml Histopaque®-1119, product nos. 10771 and 11191, respectively, Sigma-Aldrich, MO, USA) using a modification of a procedure used for chickens [35]. Trypan blue (product no. T8154, Sigma-Aldrich) was used to determine viable cell numbers. On the same day, heterophils were used to assess innate immune functions (phagocytosis and oxidative burst). See the electronic supplementary material, for detailed methods.

At week 9, 2 ml of blood were obtained and lymphocytes isolated with Histopaque® using a modification of a procedure used for chickens and wild herring gulls (*Larus argentatus*) [36]. The procedures were conducted in a sterile environment using sterile reagents and equipment. The number of viable lymphocytes was determined using trypan blue and CMI assessed with a lymphocyte proliferation assay.

Thirteen weeks after the start of the experiment, seven pigeons per treatment were randomly chosen and blood collected into tubes without anti-coagulants (Vacuette®, Greiner Bio-One). After blood clotting at room temperature, sera were collected and stored at −20°C. The pigeons were then inoculated subcutaneously with 0.5 ml of Poulvac-Newcastle iK Vaccine, inactivated LaSota Strain (Batch: 1782110A; Pfizer Australia). Blood samples were collected at two and three weeks after inoculation (weeks 15 and 16 after the start of the experiment), and sera stored at −20°C.

At the end of the experiment (week 17), all pigeons were killed with an overdose of pentobarbital sodium (Lethabarb, Virbac Animal Health, Milperra, Australia). Intestinal tracts from the junction with the gizzard to the start of the rectum were collected and stored at −20°C for later examination of the Pps.

### 2.4. Assays

Detailed information on the assays is in the electronic supplementary material, with a brief description here. Phagocytosis was measured using a modification of the methods of Rodríguez *et al*. [37] and Paredes *et al*. [38]. The number of latex beads (1.1 µm mean particle size, product no. LB11, Sigma-Aldrich) phagocytized by each heterophil (phagocytosis index: PI) and the number of heterophils that phagocytized at least one latex bead (phagocytosis percentage: PP) were counted.

The method for measuring oxidative burst was a modification of that described by Wan *et al*. [39] and He *et al*. [12,40]. 2′, 7′-Dichlorofluorescin diacetate (DCFH-DA, product no. D6883, Sigma-Aldrich) was used as a fluorescent probe and phorbol 12-myristate 13-acetate (PMA, product no. P8139, Sigma-Aldrich) as a mitogen. A change in relative fluorescent units (ΔRFU) was measured after 2 h incubation in a 37°C humidified incubator with 5% CO_2_ (Galaxy S Series, HD Scientific Supplies Pty Ltd, Edison, USA).

Lymphocyte proliferation was stimulated with three mitogens (Sigma-Aldrich). Concanavalin A (ConA, 5 µg ml^−1^) from *Canavalia ensiformis* (Type: IV-S, product no. C5275) was used as a T-lymphocyte agonist, lipopolysaccharide (LPS, 2.5 µg ml^−1^) from *Escherichia coli* 055:B5 (product no. L6529) as a B-lymphocyte agonist, and PMA (5 µg ml^−1^) as an agonist stimulating all lymphocytes [41]. After 2.5 days' incubation, alamarBlue® (BUF012B, AbD Serotec, Oxford, UK) was added to determine proliferation, and 4 and 8 h later, following its reduction by metabolites released from cells [42], absorbance was measured. Proliferation was expressed as a percentage increase of reduced alamarBlue® from mitogen-stimulated cells relative to non-stimulated cells.

Serum antibody titres to NDV were measured by a haemagglutination inhibition (HI) assay based on that of Barr & O'Rourke [43] and modified by P. Cowling (2012, personal communication). Results were expressed as a log_2_ titre value against NDV for each serum sample. Log_2_ titres ≥ 3 were considered as positive for NDV antibodies: titres < 3 were recorded as negative (P. Cowling 2012, personal communication).

Based on the method of Cornes [44], Pps were stained, numbers recorded and surface area of each Pp measured with the Fiji software program (ImageJ 1.48i, National Institutes of Health, USA).

When examining intestines, an unexpected finding was the presence of intestinal parasites, and therefore their numbers were counted.

### 2.5. Statistical analyses

For Experiment 1, data were analysed by one-way ANOVA repeated measures and the treatment means were compared with the Bonferroni's multiple comparison test (IBM® SPSS 19.0 for Windows, USA). Regression equations and 95% CIs were determined with SPSS, significance being determined from Spearman's rank correction. For Experiment 2, the data from phagocytosis, oxidative burst and lymphocyte proliferation assays and areas of Pps were analysed by one-way ANOVA and the treatment means were compared with the Tukey's *post hoc* test. The NDV HI titres and the number of parasites were analysed by a non-parametric ANOVA and the treatment means were compared with the Mann–Whitney *U* test.

## 3. Results

### 3.1. Experiment 1. Determination of endogenous nitrogen loss and maintenance nitrogen (protein) requirements

All pigeons lost BW (table 2) during each period in the metabolism cages, but they regained weight after returning to their aviaries. One pigeon was removed from the study because it lost 14.5% of BW while in the metabolism cage compared with the average of 5% for the other five pigeons. This was considered too great a loss in weight for its data to be used. The pigeon showed a negative N balance on all diets and its feed intake, irrespective of diet, was about 60% of the average feed intake of the other five pigeons. Initial and final BW of the remaining five pigeons before and after being in the metabolism cages were not significantly different among the diets (*n* = 5, *p* = 0.124 and *p* = 0.840, respectively, table 2), nor was change in BW (ΔBW) (*p* = 0.362) or ΔBW as a percentage of initial BW (*p* = 0.426).

**Table 2.** Experiment 1: Initial and final body weights (BWs) and nitrogen (N) intake, excretion and balance of pigeons fed three diets of different CP contents (mean ± s.d., *n* = 5). Different lower-case letters in the same row indicate significant difference at *p* < 0.05 using Bonferroni's multiple comparison test. N balance = (N intake − N excreted).

diet (CP)	6%	10%	14%
initial BW (g)	368.0 ± 20.2	376.0 ± 13.2	380.4 ± 13.2
final BW (g)	350.5 ± 17.6	359.9 ± 16.3	356.8 ± 10.2
N intake (mg d^−1^)	196.5 ± 25.4^a^	349.7 ± 53.5^b^	534.7 ± 55.6^c^
N excreted (mg d^−1^)	201.4 ± 34.5^a^	279.1 ± 38.4^b^	414.1 ± 52.8^c^
N balance (mg d^−1^)	−4.9 ± 29.0^a^	70.5 ± 23.2^b^	120.6 ± 14.9^c^

### 3.2. N intake, excretion and balance

Both N intake and excretion significantly (*p* < 0.001, *n* = 15) decreased as the N concentration of the diet decreased (table 2). The N retained (N intake – N excretion) was also significantly reduced when pigeons were on the 6% CP diet compared with the 10% (*p* = 0.005, *n* = 5) and 14% (*p* < 0.001, *n* = 5) CP diets (table 2).

**Figure 1.**
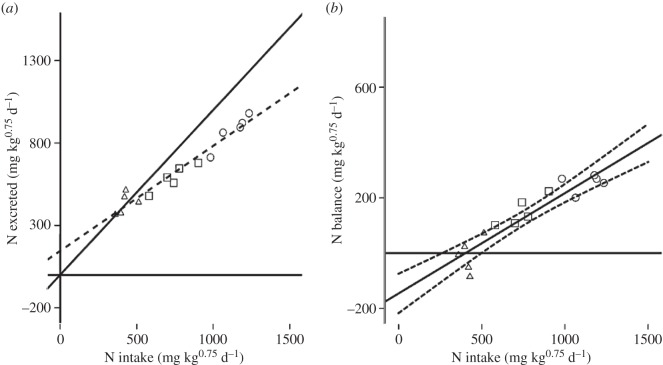
Experiment 1. (*a*) N intake (*x*-axis) versus N excreted (*y*-axis) for pigeons (*n* = 5) when fed the three CP diets (triangles = 6%, squares = 10%, circles = 14%). The broken line indicates the regression equation: *y* = 0.64*x* + 145.4 (*r^2^* = 0.951, *p* < 0.001). The solid line indicates the zero N balance line. (*b*) Relationship between N intake (*x*-axis) and N balance (*y*-axis) of pigeons when fed the three CP diets. The solid line indicates the linear regression line and the broken lines indicate the 95% CIs. Regression equation is *y* = 0.36*x* – 145.4 (*r^2^* = 0.863, *p* < 0.001).

Pigeons were in positive N balance (N excreted < N intake) when fed all the experimental diets except for two pigeons when fed the 6% CP diet (points above the solid N balance line, figure 1*a*). The ENL (±95% CIs), that is N excretion when N intake equals zero (figure 1*a*), of the pigeons was calculated to be 145.4 mg N kg^0.75^ d^−1^ (±95% CIs: 74.2–216.7) from the regression of N intake against N excreted (*y* = 0.64*x* + 145.4, *r^2^* = 0.951, *p* < 0.001), whereas MNR was calculated to be 400.6 mg N kg^0.75^ d^−1^ (±95% CIs: 204.4–597.0) from the regression of N intake against N balance (*y* = 0.36*x* − 145.4, *r^2^* = 0.863, *p* < 0.001) when the value for N balance is zero (figure 1*b*).

### 3.3. Experiment 2. Dietary protein and immune function

#### 3.3.1. Innate immunity

There was no significant difference (*p* = 0.835) in the average BW among the three treatments at week 6 when the blood samples were taken (table 3). Cell viability after Histopaque® isolation was greater than 90% at all sampling times.

**Table 3.** Experiment 2. Phagocytosis, oxidative burst (in relative fluorescent units, RFU), Newcastle disease vaccine-haemagglutination inhibition (NDV-HI) titres, Peyer's patches (Pps) and parasites in pigeons fed one of three experimental diets containing different levels of CP. Different lower-case letters in the same row indicate significant differences at *p* < 0.05 in Tukey's *post hoc* test, or for NDV-HI titres and no. of parasites/tract using Mann–Whitney *U* test at *p* < 0.05.

diet	6%	10%	14%
*innate immunity*^1^			
BW at week 6	354 ± 37.7	361 ± 28.3	360 ± 48.8
phagocytosis index (PI)^2^	301 ± 15.5^a^	678 ± 43.2^b^	669 ± 33.7^b^
phagocytosis percentage (PP)^3^	59.4 ± 2.3^a^	80.7 ± 2.1^b^	81.9 ± 1.9^b^
oxidative burst (RFU)^4^	0.6 ± 0.1^a^	1.3 ± 0.2^b^	1.2 ± 0.2^b^
*humoral immunity*^5^ (*log_2_ titre*)			
BW at week 13	359 ± 17.8	364 ± 14.0	375 ± 15.1
2 weeks post-inoculation	2.7 ± 0.6^a^	4.0 ± 0.3^ab^	4.9 ± 0.3^b^
3 weeks post-inoculation	2.3 ± 0.7^a^	3.3 ± 0.3^a^	4.9 ± 0.4^b^
*Peyer's patches* (*Pps*)^6^			
no. of Pp/tract	2.6 ± 0.4	3.5 ± 0.5	3.2 ± 0.2
surface area (mm^2^)/patch	10.7 ± 0.9	11.1 ± 0.9	13.1 ± 1.7
total surface areas (mm^2^)/tract	25.5 ± 4.4	40.2 ± 7.0	38.8 ± 2.9
no. of parasites/tract	20.5 ± 13.5^a^	6.1 ± 2.5^a^	nil^b^

^1^Mean ± s.e., *n* = 17: 6 and 10%; *n* = 16: 14% except for BW expressed as mean ± s.d.

^2^The number of latex beads phagocytized by each heterophil.

^3^The number of heterophils that phagocytized at least one latex bead (%).

^4^Calculated as (RFU_stimulated_ – RFU_non-stimulated_)/RFU_non-stimulated_.

^5^*n* = 7, HI titres non-detectable at week 13.

^6^Mean ± s.e., *n* = 14: 6 and 10%; *n* = 9: 14%.

PI of heterophils from pigeons on the 10 and 14% CP diets was two times greater (*p* < 0.001) than those on the 6% CP diet (table 3) and PP was about 25% greater (*p* < 0.001). There were no significant differences between samples from pigeons on the 10 and 14% CP diets for both PI (*p* = 0.977) and PP (*p* = 0.926).

After 2 h incubation, oxidative burst (table 3) in the pigeons fed the 6% CP diet was significantly lower than in those fed the 10% (*p* = 0.003) and 14% (*p* = 0.007) CP diets.

#### 3.3.2. Cellular-mediated immunity

At 4 and 8 h after adding alamarBlue®, all lymphocyte samples showed proliferation, irrespective of mitogens and dietary CP diets. The proliferative responses for all treatments were higher when PMA was used as mitogen, but this was not significant.

**Figure 2.**
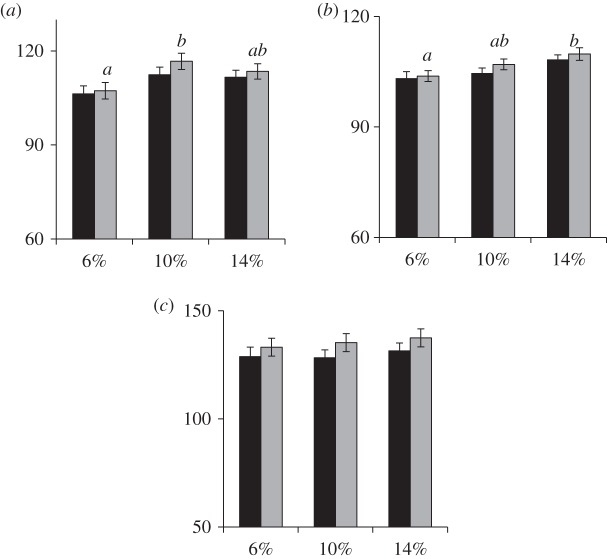
Experiment 2. Lymphocyte proliferation (% increase of mitogen-stimulated cells relative to un-stimulated cells) on *y*-axis, stimulated by (*a*) ConA at 5 µg ml^−1^, (*b*) LPS at 2.5 µg ml^−1^ and (*c*) PMA at 5 µg ml^−1^ on pigeons fed the 6, 10 and 14% crude protein (CP) diets on *x*-axis at 4 (black) and 8 (grey) hour incubation after adding alamarBlue® (mean ± s.e., *n* = 17: 6 and 10%; *n* = 16: 14%). Different lower-case letters within the same hour indicate significant differences at *p* < 0.05 using Tukey's *post hoc* test.

At 8 h after adding alamarBlue®, the ConA-stimulated lymphocytes of pigeons fed the 6% CP diet showed significantly less proliferation (*p* = 0.033) compared with the 10% CP group but not the 14% CP group (*p* = 0.224, figure 2*a*). The LPS-stimulated lymphocyte proliferation was significantly (*p* = 0.024) reduced at 8 h in the pigeons fed the 6% CP diet compared with those fed the 14% CP diet but not the 10% CP diet (*p* = 0.319, figure 2*b*). No significant difference was found among the three dietary treatments in lymphocytes stimulated with PMA although proliferation stimulated with this mitogen seemed to increase with increases in the concentrations of CP in diet (figure 2*c*).

#### 3.3.3. Humoral immunity

The pigeons had no detectable ND antibodies prior to inoculation (sampled after 13 weeks on the experimental diets, table 3). At two weeks post-inoculation, the HI titres in the pigeons fed the 6% CP diet were significantly lower (*p* = 0.012) than those in pigeons fed the 14% CP diet but there were no significant differences between those fed the 10% and those fed the 6% (*p* = 0.118) and 14% CP diets (*p* = 0.115, table 3). At three weeks post-inoculation, on the other hand, the pigeons fed the 6 and 10% CP diets had significantly lower titres than those fed the 14% CP diet (*p* < 0.02, table 3).

There were no significant differences within the same CP group between BWs at pre-inoculation and at two (*p* = 0.757) and three (*p* = 0.814) weeks post-inoculation**.**

#### 3.3.4. Peyer's patches

When dissecting the intestines for measurements of Pps, some of the pigeons were found to be infected with intestinal roundworms, and therefore the number of parasites was recorded (table 3). The pigeons fed the 6 and 10% CP diets had significantly (*p* = 0.002) higher numbers of parasites in their intestines than those fed the 14% CP diet, none of which had worms. Nine pigeons on the 6% CP diet were found to have intestinal parasites. Although ten individuals had intestinal parasites on the 10% CP diet, two of them had very small, fine parasites, which were difficult to count accurately even under the dissecting microscope, and therefore the two individuals were removed for purposes of statistical analysis. It is important to mention that none of the five pigeons used in the balance experiment or the 21 pigeons used to determine antibody production to ND had intestinal parasites.

The number of Pps ranged from one to six (*n* = 37), their surface areas ranged from 3.326 to 24.775 mm^2^ (*n* = 37), and their total surface areas ranged from 3.326 to 74.298 mm^2^ (*n* = 37). Although the values were higher in the pigeons fed the 10 and 14% CP diets than in those fed the 6% CP diet, there were no significant differences in the numbers, surface areas and total surface areas of Pps (*p* = 0.278, 0.301 and 0.120, respectively, table 3) among the three treatments.

## 4. Discussion

The ENL of granivorous pigeons was found to be in the range reported for granivorous budgerigars (*Melopsiacus undulates*) [3] and granivorous zebra finches (*Taeniopygia guttata*) [45]. The MNR was also similar to those of the budgerigar and zebra finch. However, there can be considerable variation in MNR because its value can be affected by the digestibility and quality of protein, energy and ingredients used in an experimental diet [46]; this was not relevant in these experiments as the same experimental diets were used to determine N requirements and immune functions.

Based on the MNR of 400.6 mg N kg^0.75^ d^−1^ determined in Experiment 1, the pigeons should have obtained sufficient protein from the 6% CP diet to meet their requirements and maintain N balance. Nitrogen intake of the pigeons fed the 6% CP diet in Experiment 1 averaged 196.5 ± 25.4 mg N d^−1^ (table 2), and calculating from the MNR equation, a pigeon of 365.3 g (the average BW in Experiment 1) requires 188.2 mg N d^−1^. However, two out of the five pigeons (40%) in Experiment 1 exhibited negative N balance, which suggests that this amount may not have been adequate even though BW was maintained.

Heterophils are the first leucocytes to migrate rapidly to a site of infection in order to phagocytize and destroy pathogens [9], and the ability of pigeons on the 6% CP diet to engulf particles (PI) and the number of heterophils that phagocytized particles (PP) was reduced compared with those on the 10 and 14% CP diets. Thus, their abilities to resist diseases could have been compromised by protein intake. This reduction could have been caused by alterations to the protein receptors expressed on the surface of the heterophils that recognize foreign bodies and/or a reduced phagocytic ability. Alterations in the protein enzymes responsible for changing the actin cytoskeleton, a process essential for causing the change in membrane shape required for engulfing particles [11], could also have been affected by decreased protein availability. A similar reduction in phagocytosis of sheep red blood cells by macrophages from the peritoneal exudate was reported in one-week-old broiler chickens fed a low protein (18%) diet compared with a high protein (20%) diet [25].

There have been very few studies in which the effects of dietary protein levels on phagocytosis and oxidative burst in birds were investigated. However, in protein-deficient guinea pigs, bactericidal effects of neutrophils are reduced [47], which may be a combination of reduced phagocytosis or reduced production of ROS as seen in the heterophils of the pigeons on the 6% CP diet. In insects [19,20], low protein intakes reduce bactericidal phenoloxidase production which is an analogous process in the haemocytes to oxidative burst in heterophils.

The reduction in oxidative burst activity of heterophils in the 6% CP pigeons could have been the result of reduced synthesis of PKC (a protein) and thus to a reduction in the activity of NADPH oxidase required to produce ROS and cause oxidation of DCFH-DA to the fluorescent compound DCF. Kiron *et al*. [21] have suggested that reduced intakes of protein by rainbow trout could have contributed to reduced production of lysozymes and C-reactive protein in neutrophils and macrophages, which in turn resulted in reduced immune function.

Lymphocyte proliferation mediated by ConA requires protein cytokines [48]. Concanavalin A binds non-covalently to carbohydrate groups, which are located on the cell surface [48]. This binding increases the rate of calcium entry into the cell, which causes the stimulus for DNA synthesis resulting in cell proliferation [48]. On the other hand, proliferation stimulated with LPS requires toll-like receptor 4 (TLR4), which is a leucine-rich repeat protein on the cell surface [49]. PMA (as described before) is a very powerful activator for PKC. The PKC is mainly known as an activator of T-lymphocyte proliferation [50], but can also activate B-lymphocytes [51]. Proliferation of both T- and B-lymphocytes (induced by ConA and LPS, respectively) in the 6% CP group was lower than those from pigeons on the 10 and 14% CP diets possibly due to reduced availability of amino acids for synthesis of new proteins, such as protein cytokines and TLR4 required for proliferation of T- and B-lymphocytes, respectively. That no significant differences were found in proliferation stimulated with PMA might have been due to the large variation in responses. The cause of the variation could have been due to a combination of the stimulatory functions of PMA on both T- and B-lymphocytes, but it would be impossible to explain based on this experiment.

Based on the results after six weeks on the experimental diets (phagocytosis and oxidative burst) and nine weeks (ConA- and LPS-stimulated lymphocyte proliferation), the 10% CP diet was adequate to maintain immune function. However, this was different to the effects on humoral immunity seen after 16 weeks on the experimental feeds, where there was a significant reduction in antibody titres against NDV at three weeks post-inoculation. A relatively short time of feeding laying hens (12 weeks) and a small difference in protein contents (14, 16 and 18%) have been shown to cause a significant difference in ND titres between those fed diets containing 14 or 16% protein (2.2 and 2.5 antibody titres, respectively) and those fed a diet containing 18% protein (4.2) [26]. A similar result was also found at 27 weeks in laying hens fed a 14% CP diet (2.0) compared with those fed an 18% CP diet (3.3) [29]. It is not possible to identify the specific cause of the reduced antibody titres in the pigeons on the 6 and 10% CP diets, but a reduction in the ability of B-lymphocytes to synthesize antibodies due to reduced availability of amino acids or an imbalance in amino acids [52] could have contributed; it is also possible that receptors on cell surfaces of other types of lymphocyte involved in antigen recognition or production of cytokines were reduced [21,52].

Contrary to there being no significant reduction on Pps caused by low protein intakes found in this study, eight-week-old cotton rats (*Sigmodon hispidus*) fed for six weeks with a low protein (4%) diet had two times greater relative weights (mg/g BW) of Pps than those fed a high protein (16%) diet [53]. This might have been due to the differences between rats and pigeons in developmental age or between mammals and birds. Although not statistically significant, the reduction in the number, surface area and total surface area of Pps in the pigeons fed the 6% CP diet might reflect impairment in growth caused by a lack of protein for cell division [53].

The presence of intestinal parasites was unexpected although the pigeons had not been treated with an anthelmintic before the start of the experiment. As the pigeons were randomly distributed across the three treatment groups at the start of the experiment, it was unlikely that the pigeons without parasites were all placed in the 14% CP group. Therefore, it would be expected that an equal number of the pigeons infected with intestinal parasites would be distributed across the three groups. The intestinal parasites found in the current study were *Ascaridia* spp., which are typically found in the upper part of the small intestine of birds and general symptoms include weight reduction and weakness in the host [54]: these were not observed in infected pigeons.

A defence mechanism against parasites usually involves phagocytosis and T- and B-lymphocytes [55]. The presence of parasites in the intestinal tract suggests that the pigeons were unable to induce phagocytosis or T- and B-lymphocytes against parasites; in mice, Slater & Keymer [30] showed that those fed diets containing 2 or 4% protein were unable to develop immunity to nematodes whereas those given a diet with 8% were resistant.

In this study, in spite of infection with *Ascaridia* spp*.*, the immune functions (e.g. phagocytosis, oxidative burst and ConA- and LPS-stimulated lymphocyte proliferation) in the pigeons on the 10% CP diet were not significantly reduced. The lower immune responses found in the 6% CP group were therefore most probably caused by reduced protein intake from the diet or a reduction in efficiency of nutrient absorption [56].

In poultry, digestive enzyme activity can be reduced in the presence of *Ascaridia galli* [57]. Although Walker and Farrell [58] showed that *A. galli* reduced energy and protein retention by about 20% of that of uninfected poultry, parasites do not always have an effect on reducing net absorption of amino acids [59]. Crompton & Nesheim [60] suggest that there are no consistent interactions between nutrition and parasites and contrary to the findings here in which parasite burden was greater in pigeons on the low protein diets, Permin *et al*. [54] have shown that laying hens given a diet containing 14% protein grew better and had a lower parasite burden than those given a diet containing 18% protein. It is also important to note that none of the pigeons used to assess humoral immune function were infected with parasites. Therefore, the significant reduction in ND titres observed in pigeons fed CP diets at 6 and 10% would have been due to low protein intakes and not to any interfering effects of parasites.

**Figure 3.**
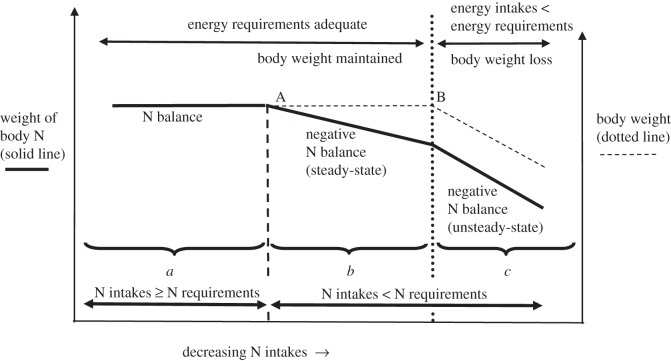
Conceptual nitrogen (protein) balance model (modified from Vogel *et al*. [61]) of body N concentrations (solid line) and BW (dotted line) against N and energy intakes in pigeons. *a*, pigeons in N balance when N and energy intakes are adequate to meet requirements; *b*, steady-state negative N balance when N intakes < N losses, however BW is maintained by adequate energy intakes; *c*, unsteady-state negative N balance when N loss relative to N intake continues to increase and/or energy intakes are unable to satisfy requirements so that tissue is lost and BW is not maintained. A, change from N balance, to steady-state negative N balance; B, change from steady-state negative N balance to unsteady-state negative N balance.

The results of this experiment show that although pigeons were not losing BW and, based on the results of the balance study (Experiment 1), were able to satisfy their nitrogen requirements for maintenance when eating the 6% CP diet, they were not able to maintain immune functions at the levels seen in the pigeons fed the 14% CP diet. The maintenance of BW in the face of N intakes at or slightly below MNR may be explained using a conceptual model (figure 3) of steady-state and unsteady-state negative N balance, which we have based on that of Vogel *et al*. [61] developed for the effects of starvation and reduced N intakes in mammals. We suggest that the pigeons eating the 6% CP diet were at or slightly to the right of point A (figure 3); that is, their N intakes were equal to or slightly below their MNR. As they were not losing BW, their energy and other nutrient intakes (besides nitrogen) were probably adequate to meet requirements; they could thus have been in steady-state negative N balance (region *b*, figure 3 as defined by Vogel *et al*. [61]). The longer they were maintained on the experimental diet, the more likely they were to shift to the right of point A thus becoming increasingly N deficient. Length of time on the experimental diets could also help to explain why the pigeons fed the 10% CP diet for 16 weeks were unable to maintain ND antibody titres to the same level as those of pigeons fed the 14% CP diet, whereas they were able to maintain lymphocyte proliferation at nine weeks.

The results of the current experiments show that an N balance study carried out for a relatively short period of two to three weeks may not accurately reflect nutrient requirements for all metabolic or physiological functions. The 10% CP diet provided about 40% more protein than the MNR, and it did not lead to reduced phagocytosis, oxidative burst or lymphocyte proliferation. It may, therefore, be advisable to measure uric acid, urea and δ^15^ N as has been suggested by Vogel *et al*. [61] when determining N requirements for different physiological functions so as to better assess N status of experimental birds.

In conclusion, caged domestic pigeons with a BW of 360 g should eat at least 3 g of protein (N intake at 14% CP, table 2) or preferably 5 g of protein (14% CP × 36 g (10% of BW)) a day in order to maintain adequate immune function. Pigeons are habitually fed about 10% of their BW but will eat more if it is available. For breeding pigeons or those subjected to stresses of racing or exposed to new diseases, requirements may be greater. The results of this experiment highlight the importance of knowing the physiological functions that can be adequately maintained by recommended nutrient requirements.

## Supplementary Material

1) Electronic supplementary material file for methodology: additional information on methodology

## Supplementary Material

2) Electronic supplementary material file for experimental diets: The detailed composition and nutritional values for the three experimental diets for pigeons

## Supplementary Material

3) Electronic supplementary material file for raw data: raw data for all the assays used in this experiment

## Data accessibility

All the data have been made available both in the electronic supplementary material and Dryad (http://dx.doi.org/10.5061/dryad.864bv).
