# Supplementary material for: Functions of innate and acquired immune system are reduced in domestic pigeons (Columba livia domestica) given a low protein diet
Source: R Soc Open Sci. 2016 Mar 23;3(3):150408. doi: 10.1098/rsos.150408 (PMC4821251; doi:10.1098/rsos.150408)
Supplement: 1) Electronic supplementary material file for methodology: additional information on methodology [file rsos150408supp1.docx]

***Additional information on methodology***

*Experiment 1 Determination of ENL and MNR*

*Metabolism cages*

The galvanised mesh (1cm^2^) sides of the metabolism cages, 0.55 m long x 0.45 m wide x 0.4 m high, were lined half-way up with Teflon sheeting into which two small holes were cut out so that the pigeons could access their feed and water attached outside the cages. Plastic-coated stainless steel wire mesh floors allowed the excreta to drop onto Teflon collecting trays and prevented the birds from coming into contact with their excreta. Two Teflon covered block-perches, 0.1 m long x 0.07 m wide x 0.07 m high, were placed in each cage.

*Sample collection*

After training to accustom the pigeons to living in the metabolism cages, they were individually housed in the cages over five days. Approximately 80ml of 4% boric acid solution (Unilab^®^, Ajax Finechem, NSW, Australia) were placed on the Teflon trays to reduce N loss. Each day, experimental feed provided was accurately weighed, and over the last three days, excreta and uneaten feed were collected each day. Excreta were mixed well with the boric acid and washed from the trays and sides of the cage with deionised water. The three daily samples were pooled for each pigeon. Samples were stored at – 20°C between and after daily sample collections.

*Experiment 2 Dietary protein intake and immune function*

*Phagocytosis*

Two hundred microlitres of the heterophil suspension (5 x 10^5^cells/ ml RPMI-1640 Medium, product no. R8755, Sigma-Aldrich) were placed in duplicate in a four-chamber slide (Lab-Tek^®^ II, Thermo Scientific, cat no. 154526, NY, USA) and incubated for 30 min in a humidified incubator at 37°C with 5% CO_2_ atmosphere (Galaxy S Series, HD Scientific Supplies Pty Ltd, Edison, USA). Then, the adhered monolayer on the slide was gently washed with 1M phosphate buffered saline (PBS) at pH 7.4 to remove non-adherent cells and 20µl of latex beads (1.1 μm mean particle size, diluted to 1% in PBS, product no. LB11, Sigma-Aldrich, MO, USA), followed by 200μl PBS were added to each chamber, gently mixed with a Pasteur pipette, and placed in the incubator described above. After 30 minutes, the slides were gently washed twice with PBS, air-dried, and stained with Wright’s stain (Amber Scientific, Australia). The first 100 heterophils observed on each chamber were examined for presence of latex beads with an oil-immersion microscope.

*Oxidative burst*

Wells for blank, non-stimulated (positive control), and phorbol 12-myristate 13-acetate-stimulated treatments (PMA, product no. P8139, Sigma-Aldrich, MO, USA) were set up in triplicate in a flat bottom, black 96-well microplate (PerkinElmer^®^, product no. 6005182, MA, USA). The wells for the blanks contained 102.5μl of RPMI-1640 Medium (product no. R8755, Sigma-Aldrich, MO, USA), those for non-stimulated cells had 100μl of 2 x 10^6^ cells/ ml with 2.5μl of media, and those for PMA-stimulated cells contained 100μl with 2.5μl of PMA (1mg/ ml dimethyl sulfoxide (DMSO), product no. D2650, Sigma-Aldrich, MO, USA). Then, 2.5μl of 2’, 7’-dichlorofluorescin diacetate (DCFH- DA, product no. D6883, Sigma-Aldrich, MO, USA; 1mg of DCFH-DA/ ml absolute ethanol) were added to all wells (total volume: 105μl/ well). The plate was covered with aluminium foil to prevent any exposure to light, and placed in a 37°C humidified incubator with 5% CO_2_. After 2h incubation, fluorescence intensity (relative fluorescence unit: RFU) was measured at 485-nm/530-nm (excitation/emission wavelengths) in a fluorescence microplate reader (EnSpire, PerkinElmer^®^, MA, USA).

*Lymphocyte proliferation*

This assay was conducted in a sterile environment in a laminar flow cabinet (Clemco, Australia) at all times. The RPMI-1640 Medium used here contained penicillin-streptomycin (10,000 units penicillin and 10 mg streptomycin per ml in 0.9% NaCl, product no. P0781, Sigma-Aldrich, NSW, Australia) to avoid microbial contamination and proliferation.

Wells for blank, positive control and treatment with the mitogens were set up in triplicate in a sterile, flat bottom, clear 96-well microplate (Falcon^®^, Becton Dickinson Labware, MA, USA). The blank wells contained only 100μl of RPMI-1640 Medium, positive control wells contained 50μl of medium and 50μl of 5 x 10^6^ cells/ ml medium. Before each mitogen (Sigma-Aldrich, MO, USA) was dispensed into the microplate, concanavalin A (ConA, product no. C5275) and lipopolysaccharide (LPS, product no. L6529) were dissolved in sterile medium, and PMA was dissolved in sterile DMSO to a concentration of 1mg/ ml. Then, a 50μl aliquot (ConA and PMA at 10μg/ml; LPS at 5μg/ml) of each of these mitogens was added to each of 3 wells per sample, followed by 50μl of 5 x 10^6^ cells/ ml RPMI-1640 added to make up the final volume of 100μl in each well. The plate was then placed in a 37°C humidified incubator with 5% CO_2_ for 2.5 days.

After incubation, alamarBlue^®^ (BUF012B, AbD Serotec, Oxford, UK) was prepared according to the manufacturer’s procedure. One hundred microlitres of alamarBlue^®^ were added into each well. The plate was again incubated until absorbance was measured at 4 and 8h with a microplate reader (EnSpire, PerkinElmer^®^, MA, USA) at two wavelengths, 570- nm and 600-nm.

The incubation temperature used for the assays was 37 °C, which is lower than the avian body temperature of 41 °C when active [63]. However, the body temperature at rest is 38.5 °C in birds [63], which was more likely the condition of the pigeons kept here in the aviaries. Comparisons between treatments were not made based on absolute values, and therefore, the temperature used in this experiment should not affect the results obtained here.

*Newcastle disease vaccine-haemagglutination inhibition (NDV-HI) assay*

Twenty-five microlitres of PBS were added to each well of a V-shaped 96 well microtitre plate. For the sample plate, 25μl of a pigeon serum sample were added to the first well of the row A, and were serially diluted (1 in 2 dilution) from left to right. The extra 25μl in the final wells were removed and discarded. This was repeated depending on the number of samples. In the control plate (does not contain pigeon serum samples), 25μl of a positive serum of known ND antibody titre (+S; positive control) were added to each of three wells, 25μl of a specific pathogen free (SPF) serum negative for ND antibodies (negative control) were added to a single well, 25μl of the diluted antigen (DA; the antigen to NDV V4 prepared in SPF chicken eggs) were added to further three wells: they were then serially diluted as described above. The last row of the plate contained 50μl per well of PBS (cell controls). Twenty-five microlitres of DA were added to each well except for those already containing DA and the cell controls. For the wells containing DA, 25μl of PBS were added instead to make up to the same volume. The plates were covered and placed on a microshaker for 30 sec. The covered plates were incubated at 4°C for 30 min and then 25μl of a freshly prepared 1% solution of chicken erythrocytes in PBS at pH 7.4 were added to each well (both sample and control plates). The plates were again placed on the shaker for 30 sec, and incubated under the same conditions as described above or until discrete buttons were formed in the cell controls. The presence and size of pellets were then assessed by eye. The known +S was checked to ensure that it gave a titre of 10^7^ ± 1, the SPF negative serum wells that they contained no pellets, and the DA wells that they gave a titre of 10^3^ ± 1.

*Peyer’s patches (Pps)*

After the intestinal tracts of pigeons were defrosted overnight at 4°C, the external surface was washed in running tap water, and inner surface (lumen) was flushed using a syringe attached to a thick needle (14G) until all mucous and blood had been washed off. The intestinal tracts were then slit open along the longitudinal axis where the mesentery joins the tracts. The specimens were then placed in a plastic container and covered with 5% acetic acid solution for 24 hours at 4°C. The specimens were washed in water, stained with 0.5% polychrome methylene blue (Amber Scientific, Australia) for one minute, and rinsed with water to remove excess stain. They were then placed in a bag containing water for one hour before being examined. The intestinal tracts were digitally photographed with a standard scale in each photograph. After uploading to a computer, the surface areas of the Pps were measured with the Fiji software program (ImageJ 1.48i, National Institutes of Health, USA).
