# Supplementary material for: Functions of innate and acquired immune system are reduced in domestic pigeons (Columba livia domestica) given a low protein diet
Source: R Soc Open Sci. 2016 Mar 23;3(3):150408. doi: 10.1098/rsos.150408 (PMC4821251; doi:10.1098/rsos.150408)
Supplement: 2) Electronic supplementary material file for experimental diets: The detailed composition and nutritional values for the three experimental diets for pigeons [file rsos150408supp2.pdf]

3150 Great Eastern Hwy
Glen Forrest
Western Australia 6071
p: +61 8 9298 8111
F: +61 8 9298 8700
Email: info@specialtyfeeds.com

Diet SF11-068

6% Protein Pigeon Pellets

A low protein Semi pure diet for pigeons based on customer specifications

Calculated Nutritional Parameters

Protein	6.00%
Total Fat	4.20%
Crude Fibre	4.70%
Acid Detergent Fibre	4.70%
Metabolisable Energy	15 MJ / Kg
% Total Calculated Digestible Energy From Protein	7.90%
% Toatal Calculated Digestible Energy From Lipids	9.80%

Diet Form and Features

- The diet is manufactured as an 4 mm diameter cube 10 - 30 mm long.
- Packaging is in 5Kg oxygen impermeable bags under nitrogen, placed in cardboard cartons for protection during transit.

Feeding Recommendations

This diet should be introduced gradually into the animals ration over at least a 14 day period.

Ingredients

Starch	765 g/Kg
Soy Protein Isolate	61 g/Kg
Cellulose	50 g/Kg
Soya Bean Oil	40 g/Kg
Mono Calcium Phosphate	20.7 g/Kg
Calcium Carbonate	14.8 g/Kg
Potassium Dihydrogen Phosphate	10 g/Kg
Pigeon Vitamins	10 g/Kg
Salt	6 g/Kg
DL Methionine	6 g/Kg
Lysine	5 g/Kg
Choline Chloride 60%	5 g/Kg
Glycine	4 g/Kg
Pigeon Trace Minerals	1.6 g/Kg
Potassium Chloride	1 g/Kg

Calculated Amino Acids	
Valine	0.24%
Leucine	0.43%
Isoleucine	0.24%
Threonine	0.20%
Methionine	0.66%
Cystine	0.06%
Lysine	0.72%
Phenylanine	0.28%
Tyrosine	0.21%
Tryptophan	0.06%

Calculated Total Minerals	
Calcium	0.86%
Phosphorous	0.79%
Magnesium	0.04%
Sodium	0.34%
Potassium	0.37%
Sulphur	0.19%
Iron	290 mg/Kg
Copper	11 mg/Kg
Iodine	1.7 mg/Kg
Manganese	106 mg/Kg
Cobalt	1.2 mg/Kg
Zinc	170 mg/Kg
Molybdenum	4.7 mg/Kg
Selenium	0.1 mg/Kg
Cadmium	No data

Calculated Total Vitamins	
Vitamin A (Retinol)	4 500 IU/Kg
Vitamin D (Cholecalciferol)	450 IU/Kg
Vitamin E (a Tocopherol acetate)	52 mg/Kg
Vitamin K (Menadione)	1.5 mg/Kg
Vitamin C (Ascorbic acid)	150 mg/Kg
Vitamin B1 (Thiamine)	15 mg/Kg
Vitamin B2 (Riboflavin)	15 mg/Kg
Niacin (Nicotinic acid)	51 mg/Kg
Vitamin B6 (Pryridoxine)	7.8 mg/Kg
Pantothenic Acid	20 mg/Kg
Biotin	600 ug/Kg
Folic Acid	6.1 mg/Kg
Inositol	0.3 mg/Kg
Vitamin B12 (Cyancobalamin)	16.5 ug/Kg
Choline	3 220 mg/Kg

Calculated Fatty Acid Composition	
Myristic Acid 14:0	Trace
Palmitic Acid 16:0	0.40%
Stearic Acid 18:0	0.15%
Oleic Acid 18:1	0.90%
Gadoleic Acid 20:1	0.01%
Linoleic Acid 18:2 n6	2.04%
a Linolenic Acid 18:3 n3	0.27%
Total n3	0.27%
Total n6	2.04%
Total Mono Unsaturated Fats	0.93%
Total Poly Unsaturated Fats	2.31%
Total Saturated Fats	0.57%

Calculated data uses information from typical raw material composition. It could be expected that individual batches of diet will vary from this figure.

We are happy to provide full calculated nutritional information for all of our products, however we would like to emphasise that these diets have been specifically designed for manufacture by Specialty Feeds.

This Feedstuffs does not contain restricted animal material.

Specialty Feeds
3150 Great Eastern Hwy
Glen Forrest
Western Australia 6071
p: +61 8 9298 8111
F: +61 8 9298 8700
Email: info@specialtyfeeds.com

Diet SF11-069 — 10% Protein Pigeon Pellets

A medium protein semi pure diet for pigeons based on customer specifications

Calculated Nutritional Parameters

Protein	10.00%
Total Fat	4.40%
Crude Fibre	4.70%
Acid Detergent Fibre	4.70%
Metabolisable Energy	15 MJ / Kg
% Total Calculated Digestible Energy From Protein	12.90%
% Total Calculated Digestible Energy From Lipids	9.80%

Diet Form and Features

- The diet is manufactured as an 4 mm diameter cube 10 - 30 mm long.
- Packaging is in 5kg oxygen impermeable bags, vacuum sealed under nitrogen, placed in cardboard cartons for protection during transit.

Feeding Recommendations

This diet should be introduced gradually into the animals ration over at least a 14 day period.

Ingredients

Starch	725 g/Kg
Soy Protein Isolate	101 g/Kg
Cellulose	50 g/Kg
Soya Bean Oil	40 g/Kg
Mono Calcium Phosphate	20.7 g/Kg
Calcium Carbonate	14.8 g/Kg
Potassium Dihydrogen Phosphate	10 g/Kg
Pigeon Vitamins	10 g/Kg
Salt	6 g/Kg
DL Methionine	6 g/Kg
Lysine	5 g/Kg
Choline Chloride 60%	5 g/Kg
Glycine	4 g/Kg
Pigeon Trace Minerals	1.6 g/Kg
Potassium Chloride	1 g/Kg

Calculated Amino Acids	
Valine	0.44%
Leucine	0.76%
Isoleucine	0.44%
Threonine	0.36%
Methionine	0.72%
Cystine	0.10%
Lysine	1.00%
Phenylanine	0.50%
Tyrosine	0.40%
Tryptophan	0.10%

Calculated Total Minerals	
Calcium	0.90%
Phosphorous	0.80%
Magnesium	0.04%
Sodium	0.40%
Potassium	0.40%
Sulphur	0.20%
Iron	290 mg/Kg
Copper	11 mg/Kg
Iodine	1.7 mg/Kg
Manganese	106 mg/Kg
Cobalt	1.2 mg/Kg
Zinc	170 mg/Kg
Molybdenum	4.7 mg/Kg
Selenium	0.1 mg/Kg

Calculated Total Vitamins	
Vitamin A (Retinol)	4 500 IU/Kg
Vitamin D (Cholecalciferol)	450 IU/Kg
Vitamin E (a Tocopherol acetate)	52 mg/Kg
Vitamin K (Menadione)	1.5 mg/Kg
Vitamin C (Ascorbic acid)	150 mg/Kg
Vitamin B1 (Thiamine)	15 mg/Kg
Vitamin B2 (Riboflavin)	15 mg/Kg
Niacin (Nicotinic acid)	50 mg/Kg
Vitamin B6 (Pryridoxine)	7.8 mg/Kg
Pantothenic Acid	20 mg/Kg
Biotin	600 ug/Kg
Folic Acid	6 mg/Kg
Inositol	0.3 mg/Kg
Vitamin B12 (Cyancobalamin)	20 ug/Kg
Choline	3 220 mg/Kg

Calculated Fatty Acid Composition	
Myristic Acid 14:0	Trace
Palmitic Acid 16:0	0.40%
Stearic Acid 18:0	0.15%
Oleic Acid 18:1	0.90%
Gadoleic Acid 20:1	0.01%
Linoleic Acid 18:2 n6	2.04%
a Linolenic Acid 18:3 n3	0.27%
Total n3	0.27%
Total n6	2.04%
Total Mono Unsaturated Fats	0.93%
Total Poly Unsaturated Fats	2.31%
Total Saturated Fats	0.57%

Calculated data uses information from typical raw material composition. It could be expected that individual batches of diet will vary from this figure.

We are happy to provide full calculated nutritional information for all of our products, however we would like to emphasise that these diets have been specifically designed for manufacture by Specialty Feeds.

This Feedstuffs does not contain restricted animal material.

3150 Great Eastern Hwy
Glen Forrest
Western Australia 6071
p: +61 8 9298 8111
F: +61 8 9298 8700
Email: info@specialtyfeeds.com

Diet SF11-070

14% Protein Pigeon Pellets

A medium protein semi pure diet for pigeons based on customer specifications

Calculated Nutritional Parameters

Protein	14.00%
Total Fat	4.40%
Crude Fibre	4.70%
Acid Detergent Fibre	4.70%
Metabolisable Energy	15 MJ / Kg
% Total Calculated Digestible Energy From Protein	17.80%
% Total Calculated Digestible Energy From Lipids	9.70%

Ingredients

Starch	680 g/Kg
Soy Protein Isolate	146 g/Kg
Cellulose	50 g/Kg
Soya Bean Oil	40 g/Kg
Mono Calcium Phosphate	20.7 g/Kg
Calcium Carbonate	14.8 g/Kg
Potassium Dihydrogen Phosphate	10 g/Kg
Pigeon Vitamins	10 g/Kg
Salt	6 g/Kg
DL Methionine	6 g/Kg
Lysine	5 g/Kg
Choline Chloride 60%	5 g/Kg
Glycine	4 g/Kg
Pigeon Trace Minerals	1.6 g/Kg
Potassium Chloride	1 g/Kg

Diet Form and Features

- The diet is manufactured as an 4 mm diameter cube 10 - 30 mm long.
- Packaging is in 5kg oxygen impermeable bags, vacuum sealed under nitrogen, placed in cardboard cartons for protection during transit.

Feeding Recommendations

This diet should be introduced gradually into the animals ration over at least a 14 day period.

Calculated Amino Acids	
Valine	0.63%
Leucine	1.09%
Isoleucine	0.63%
Threonine	0.52%
Methionine	0.78%
Cystine	0.14%
Lysine	1.24%
Phenylanine	0.70%
Tyrosine	0.50%
Tryptophan	0.15%

Calculated Total Minerals	
Calcium	0.90%
Phosphorous	0.80%
Magnesium	0.04%
Sodium	0.40%
Potassium	0.40%
Sulphur	0.20%
Iron	290 mg/Kg
Copper	11 mg/Kg
Iodine	1.7 mg/Kg
Manganese	106 mg/Kg
Cobalt	1.2 mg/Kg
Zinc	170 mg/Kg
Molybdenum	4.7 mg/Kg
Selenium	0.1 mg/Kg

Calculated Total Vitamins	
Vitamin A (Retinol)	4 500 IU/Kg
Vitamin D (Cholecalciferol)	450 IU/Kg
Vitamin E (a Tocopherol acetate)	52 mg/Kg
Vitamin K (Menadione)	1.5 mg/Kg
Vitamin C (Ascorbic acid)	150 mg/Kg
Vitamin B1 (Thiamine)	15 mg/Kg
Vitamin B2 (Riboflavin)	15 mg/Kg
Niacin (Nicotinic acid)	50 mg/Kg
Vitamin B6 (Pryridoxine)	7.8 mg/Kg
Pantothenic Acid	20 mg/Kg
Biotin	600 ug/Kg
Folic Acid	6 mg/Kg
Inositol	0.3 mg/Kg
Vitamin B12 (Cyancobalamin)	20 ug/Kg
Choline	3 220 mg/Kg

Calculated Fatty Acid Composition	
Myristic Acid 14:0	Trace
Palmitic Acid 16:0	0.40%
Stearic Acid 18:0	0.15%
Oleic Acid 18:1	0.90%
Gadoleic Acid 20:1	0.01%
Linoleic Acid 18:2 n6	2.04%
a Linolenic Acid 18:3 n3	0.27%
Total n3	0.27%
Total n6	2.04%
Total Mono Unsaturated Fats	0.93%
Total Poly Unsaturated Fats	2.31%
Total Saturated Fats	0.57%

Calculated data uses information from typical raw material composition. It could be expected that individual batches of diet will vary from this figure.

We are happy to provide full calculated nutritional information for all of our products, however we would like to emphasise that these diets have been specifically designed for manufacture by Specialty Feeds.

This Feedstuffs does not contain restricted animal material.
